# Biotrauma during ultra-low tidal volume ventilation and venoarterial extracorporeal membrane oxygenation in cardiogenic shock: a randomized crossover clinical trial

**DOI:** 10.1186/s13613-021-00919-0

**Published:** 2021-08-28

**Authors:** Laura Amado-Rodríguez, Cecilia Del Busto, Inés López-Alonso, Diego Parra, Juan Mayordomo-Colunga, Miguel Arias-Guillén, Rodrigo Albillos-Almaraz, Paula Martín-Vicente, Cecilia López-Martínez, Covadonga Huidobro, Luigi Camporota, Arthur S. Slutsky, Guillermo M. Albaiceta

**Affiliations:** 1 Unidad de Cuidados Intensivos Cardiológicos, Hospital Universitario Central de Asturias, Avda de Roma s/n, 33011 Oviedo, Spain; 2 Instituto de Investigación Sanitaria del Principado de Asturias, Oviedo, Spain; 3 Centro de Investigación Biomédica en Red (CIBER)-Enfermedades Respiratorias, Instituto de Salud Carlos III, Madrid, Spain; 4 Unidad de Cuidados Intensivos Pediátricos, Hospital Universitario Central de Asturias, Oviedo, Spain; 5 Servicio de Neumología, Hospital Universitario Central de Asturias, Oviedo, Spain; 6 Departamento de Biología Funcional, Instituto Universitario de Oncología del Principado de Asturias (IUOPA), Universidad de Oviedo, Oviedo, Spain; 7 Department of Adult Critical Care, Guy’s and St Thomas’ NHS Foundation Trust, Health Centre for Human and Applied Physiological Sciences, King’s College, London, UK; 8 Li Ka Shing Knowledge Institute, St Michael’s Hospital, Toronto, Canada

**Keywords:** Extracorporeal membrane oxygenation, Ventilator-induced lung injury, Mechanical ventilation, Pulmonary oedema, Respiratory mechanics

## Abstract

**Background:**

Cardiogenic pulmonary oedema (CPE) may contribute to ventilator-associated lung injury (VALI) in patients with cardiogenic shock. The appropriate ventilatory strategy remains unclear. We aimed to evaluate the impact of ultra-low tidal volume ventilation with tidal volume of 3 ml/kg predicted body weight (PBW) in patients with CPE and veno–arterial extracorporeal membrane oxygenation (V–A ECMO) on lung inflammation compared to conventional ventilation.

**Methods:**

A single-centre randomized crossover trial was performed in the Cardiac Intensive Care Unit (ICU) at a tertiary university hospital. Seventeen adults requiring V–A ECMO and mechanical ventilation due to cardiogenic shock were included from February 2017 to December 2018. Patients were ventilated for two consecutive periods of 24 h with tidal volumes of 6 and 3 ml/kg of PBW, respectively, applied in random order. Primary outcome was the change in proinflammatory mediators in bronchoalveolar lavage fluid (BALF) between both ventilatory strategies.

**Results:**

Ventilation with 3 ml/kg PBW yielded lower driving pressures and end-expiratory lung volumes. Overall, there were no differences in BALF cytokines. Post hoc analyses revealed that patients with high baseline levels of IL-6 showed statistically significant lower levels of IL-6 and IL-8 during ultra-low tidal volume ventilation. This reduction was significantly proportional to the decrease in driving pressure. In contrast, those with lower IL-6 baseline levels showed a significant increase in these biomarkers.

**Conclusions:**

Ultra-low tidal volume ventilation in patients with CPE and V–A ECMO may attenuate inflammation in selected cases. VALI may be driven by an interaction between the individual proinflammatory profile and the mechanical load overimposed by the ventilator.

*Trial registration* The trial was registered in ClinicalTrials.gov (identifier NCT03041428, Registration date: 2nd February 2017).

**Supplementary Information:**

The online version contains supplementary material available at 10.1186/s13613-021-00919-0.

## Background

Distribution of tidal volume (Vt) within lung parenchyma determines the regional distribution of forces experienced by the tissue. When end-inspiratory stretch is high or functional residual capacity is low, leading to recruitment/de-recruitment phenomena, excessive mechanical loads may promote tissue damage and inflammation [1]. In patients with the acute respiratory distress syndrome (ARDS), ventilator-associated lung injury (VALI) contributes to lung inflammation and is one major determinant of outcomes [2]. Calfee et al. identified two ARDS subphenotypes that may benefit from different ventilatory approaches, according to their proinflammatory profile [3].

In spite of different pathophysiological mechanisms, alveolar flooding caused by hydrostatic mechanisms in patients with cardiogenic pulmonary oedema (CPE) may produce similar respiratory system mechanics to those observed in patients with ARDS [4, 5]. A recent report illustrates that patients with congestive heart failure are exposed to driving and plateau pressures similar to those with ARDS [6]. However, the effects of this mechanical load on regional inflammation in patients with CPE, in which the inflammatory response may not be so activated as in ARDS, have not been studied.

Reduced tidal volume is the mainstay of the ventilatory management of ARDS [7], where decreases in driving pressures have been related to lower risk of death, with the lower threshold for this benefit still unclear. Low tidal volumes may decrease alveolar ventilation, increasing CO_2_ levels, and produce atelectasis and patient-ventilator dyssynchronies [8]. Several authors have proposed the use of ultra-low tidal volume strategies with tidal volumes of 3 ml/kg, predicted body weight (PBW) [9, 10] and extracorporeal gas exchange to remove CO_2_ and possibly improve oxygenation [11]. Veno-venous extracorporeal membrane oxygenation [9] permits this ventilatory strategy and can lead to better outcomes in patients with very severe ARDS [10, 12]. Patients with CPE due to severe ventricular dysfunction may require extracorporeal cardiopulmonary support with veno-arterial ECMO (V–A ECMO) [13] and may also benefit from ultra-low tidal volume strategies, assuming a similar respiratory mechanics and risk of VALI as ARDS patients do, due to alveolar flooding.

We conducted a prospective study aimed to evaluate changes in lung inflammation in response to an ultra-low tidal volume strategy with tidal volume of 3 ml/kg PBW in patients with CPE who were on V–A ECMO.

## Materials and methods

### Study design

This single-centre, prospective, randomized, cross-over trial was registered in Clinicaltrials.gov (identifier NCT03041428) and performed according to CONSORT statement (CONSORT checklist available as Additional file 1). All procedures performed were in accordance with the ethical standards of the institutional and/or national research committee (Comité de Ética de la Investigación del Principado de Asturias, REF 22/17) and with the 1964 Helsinki Declaration and its later amendments. Informed consent was obtained from each patients’ next of kin.

### Patients

From February 2017 to December 2018, all mechanically ventilated patients receiving V–A ECMO in the Cardiac Intensive Care Unit at Hospital Universitario Central de Asturias were screened. Inclusion criteria, other than mechanical ventilation and V–A ECMO support, were: cardiogenic shock (defined as systemic hypoperfusion with systolic blood pressure below 90 mmHg in spite of fluid resuscitation and inotropes, evidence of distant organ failure—defined as Organ-Specific SOFA score ≥ 2 [14]—and/or cardiac index < 2.2 l/min/m^2^, and corresponding to Stages C to D in the SCAI consensus definition [15]) and CPE (defined as impaired gas exchange with a PaO_2_/FiO_2_ lower than 300, bilateral infiltrates in chest X-ray with pulmonary capillary wedge pressures (PCP) > 18 mmHg and/or echocardiographic signs of congestive heart failure). Exclusion criteria were age < 18-year, immunosuppression, history of chronic respiratory diseases, known or suspected acute lung injury from other causes (pneumonia, atelectasis, massive pleural effusion), haemodynamic instability refractory to therapy, do-not resuscitate orders or a terminal condition. Intraaortic balloon pump was inserted via femoral artery in all the cases [16]. Patients were followed up to hospital discharge.

### Outcomes

The primary endpoint was the bronchoalveolar lavage fluid (BALF) interleukin (IL)-6 concentration after each ventilatory strategy. Additionally, a set of inflammatory cytokines were determined in the obtained samples. Secondary outcomes were the impact of the ventilatory strategy on respiratory mechanics and on haemodynamic variables. Planned post hoc analyses included comparisons of subgroups according to driving or plateau pressures or IL-6 levels during conventional protective ventilation (6 ml/kg PBW).

### Intervention and measurements

PBW was calculated as 45.5 + [0.91 (centimetres of height − 152.4)] for females; and 50 + [0.91 (centimetres of height − 152.4)] for males. Before inclusion, patients were ventilated with tidal volumes of 6–8 ml/kg PBW and plateau pressures below 28 cmH_2_O. After inclusion, patients were ventilated using a constant-flow, volume-controlled mode for 24 h with a tidal volume of 6 or 3 ml/kg PBW, applied in random order. After the first 24 h in the initial strategy, tidal volume was modified by 1 ml/kg PBW per hour until it achieved the target for the consecutive ventilatory strategy. Closed envelopes containing the ventilation sequence were generated before the start of the enrolment period, using a random, computer-generated list. All other ventilatory and ECMO settings were selected by the physician in charge. No other ventilatory strategies (prone position, recruitment manoeuvers) or inhaled vasodilators were allowed during the trial. Spontaneous breathing during the study period was avoided using muscle relaxants or deep sedation. Propofol was used as sedative in all the cases.

After 24 h on each ventilatory strategy, gas exchange and ventilatory and haemodynamic measurements were collected. Arterial blood gases were drawn from an arterial line placed in the upper limb contralateral to the ECMO arterial insertion. Respiratory system compliance, end-expiratory lung volume (EELV) and lung strain (estimated as the ratio between Vt and EELV) were calculated as previously described [17]. In patients with a Swan–Ganz catheter, cardiac output and pulmonary pressures were also measured.

Bronchoalveolar lavage was performed after 24 h on each strategy as previously described [17]. A catheter was inserted into the endotracheal tube beyond the distal end of the tube and placed in wedge position. The lung was lavaged with up to three 20-ml aliquots of sterile saline. The volume collected was filtered through sterile gauze, centrifuged at 1500 rpm for 15 min to remove cells, and the supernatants were filtered through 70 μm strainers and then stored at − 80 °C for subsequent analysis. Concentrations of interferon (IFN)-α2, IFN-γ, IL-1β, IL-6, monocyte chemoattractant protein 1 (MCP-1), tumour necrosis factor (TNF)-α, IL-8, IL-10, IL-12p70, IL-17A, IL-18, IL-23 and IL-33 in BALF were measured in all samples using a multiplexed, flow-cytometry assay (LegendPLEX assay panel, BioLegend, USA), following the manufacturer’s instructions.

Patients were classified as hyperinflamed or non-hyperinflamed based on IL-6 concentration on BALF during ventilation with 6 ml/kg PBW. Using data from a previous study [17], an optimal cut-off point of 680 pg/ml to discriminate between patients with and without ventilation-related lung inflammation was identified (Additional file 2: Figure S1A, B).

### Sample size calculation

Based on the BALF IL-6 levels measured in a previous study in patients with ARDS and low strain [17], the study was designed to detect a difference of 100 pg/ml in the change of IL-6 levels (matched pairs), with a standard deviation of 100 pg/ml. With 95% power and a 5% type-I error, the required sample size was 17 patients.

### Statistical analysis

All data are shown as median (interquartile range). Data obtained during ventilation with 6 and 3 ml/kg PBW were compared using a Wilcoxon test for paired data. Cytokine concentrations were log-transformed for independent comparisons. Differences in cytokines between subgroups were studied using an analysis of covariance (ANCOVA) to avoid bias due to regression to the mean [18]. Correlations among cytokines were calculated using Pearson’s correlation coefficients. When appropriate, these correlation coefficients were compared using the Fisher’s r-to-Z transformation. A hierarchical clustering algorithm was applied to the Euclidean distances between these correlations to identify groups of immune mediators with similar behaviour. All *p* values were reported, and those below 0.05 considered significant. All the analyses were performed in R (version 3.5.1).

## Results

**Fig. 1:**
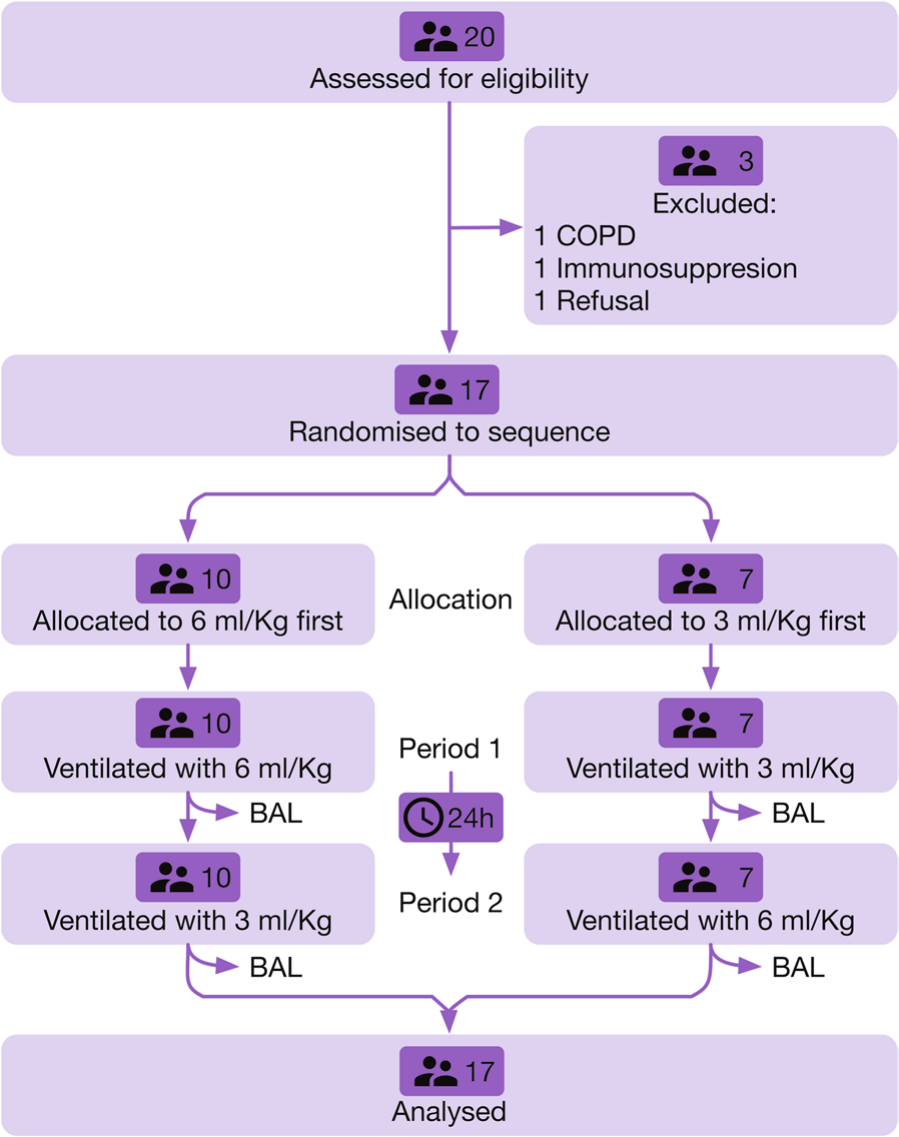
CONSORT flow diagram of the study. *COPD* chronic obstructive pulmonary disease, *BAL* bronchoalveolar lavage

Twenty patients met inclusion criteria; 3 were excluded, leaving 17 patients recruited for analysis (Fig. 1). Median age was 59 (53–65) years; 12 (70%) were male. ECMO was implanted on the first 48 h after meeting shock criteria in all the patients. Median time from onset of V–A ECMO to study inclusion was 1 (0–1) day. Baseline and clinical characteristics of all patients, including causes of cardiogenic shock, according to initial ventilatory strategy are shown in Additional file 2: Table S1. ECMO arterial insertion was central in 11 cases and peripheral in the remaining 6. None of the included patients had an open chest. During the study, all patients received vasoactive support with noradrenaline and dobutamine.

Ten patients were randomized to initial ventilation with Vt 6 ml/kg PBW and the remaining 7 with 3 ml/kg PBW. All patients were receiving neuromuscular blocking agents. Absence of spontaneous breathing was confirmed by inspection of patients’ chest and ventilator waveforms during the study period. Two patients required renal replacement therapy during the study. There were no deviations from the ventilatory protocol, and cross-over was performed in all the patients. Extracorporeal support was maintained for 8 (7–14) days, and mechanical ventilation for 15 (8–25) days. ICU and hospital mortality rates were 35% and 41%, respectively.

### Respiratory and circulatory effects of ultra-low tidal volume ventilation

Ultra-low tidal volume ventilation was well tolerated by all patients. SOFA scores were not modified by the ventilatory strategy (7.5 [6–9] vs. 7 [5–11] with Vt of 6 and 3 ml/kg PBW, respectively, *p* = 0.56). Fluid balance, urine output, renal function and haemoglobin values were not significantly different between ventilatory strategies (Table 1).

**Table 1:** Respiratory mechanics and gas exchange during ventilation with tidal volumes of 6 and 3 ml/kg PBW

	6 ml/kg PBW	3 ml/kg PBW	*p*
Tidal volume (ml)	400 (378–443)	215 (190–225)	
Respiratory rate (min^−1^)	12 (10–13)	12 (10–14)	0.096
Plateau pressure (cmH_2_O)	18 (17–20)	15 (13–17)	0.003
PEEP (cmH_2_O)	6 (5–7)	8 (6–8)	0.034
Driving pressure (cmH_2_O)	12 (10–14)	7 (6–9)	0.003
Respiratory system compliance (ml/cmH_2_O)	35 (29–40)	27 (23–32)	0.013
EELV (ml)	904 (664–1043)	538 (494–827)	0.024
Strain	0.50 (0.43–0.59)	0.38 (0.24–0.50)	0.529
PaO_2_ (mmHg)	100 (86–111)	97 (80–120)	0.782
PaCO_2_ (mmHg)	36 (32–37)	39 (36–44)	0.074
pH	7.45 (7.43–7.50)	7.40 (7.34–7.45)	0.027
FiO_2_	0.40 (0.35–0.50)	0.40 (0.35–0.50)	0.635
F_ECMO_O_2_	0.65 (0.60–0.80)	0.70 (0.60–0.80)	0.283
ECMO blood flow (l/min)	3.1 (2.9–3.7)	3.3 (2.9–3.5)	0.327
ECMO sweep flow (l/min)	3.0 (2.38–5.00)	5.0 (3.5–6.5)	0.004
Fluid balance (l)	0.736 (-0.19–2.34)	1.725 (508–3.165)	0.377
Urine output (l)	1.57 (0.85–2.88)	1.18 (0.88–2.16)	0.206
Creatinine (mg/dl)	1.12 (0.80–1.41)	1.22 (0.79–2.11)	0.804
Urea (mg/dl)	58 (33.5–77.5)	57 (40.25–79.75)	0.974
Use of RRT (*n*)	2	2	1
Haemoglobin (g/dl)	9.1 (8.85–10.05)	8.8 (8.47–9.47)	0.083

Baseline kidney function and haemoglobin values are also depicted. Data are expressed as median (interquartile range)

*PEEP* positive end-expiratory pressure, *RRT*: renal replacement therapy, *EELV*: end-expiratory lung volume. EELV and strain were measured in only nine patients

Ventilation with 3 ml/kg PBW yielded lower plateau and driving pressures (Table 1). Respiratory rate was not modified. There was a small, but significant, increase in positive end-expiratory pressure (PEEP) and a significant decrease in compliance with ultra-low tidal volume ventilation. Auto-PEEP was not detected in any patient. EELV was measured with both ventilatory settings in 9 patients, decreasing with ultra-low tidal volume ventilation. Differences in strain between strategies were negligible.

There were no significant differences in PaO_2_ or PaCO_2_ between strategies, with a significant but not clinically relevant decrease in pH. Oxygen fraction in ventilator and ECMO membrane were also similar between the ventilatory settings. Sweep gas flow was increased during ultra-low tidal volume ventilation. There were no significant effects of ultra-low tidal volume ventilation on haemodynamic parameters (Additional file 2: Table S2).

### Lung inflammation during ultra-low tidal volume ventilation

**Fig. 2:**
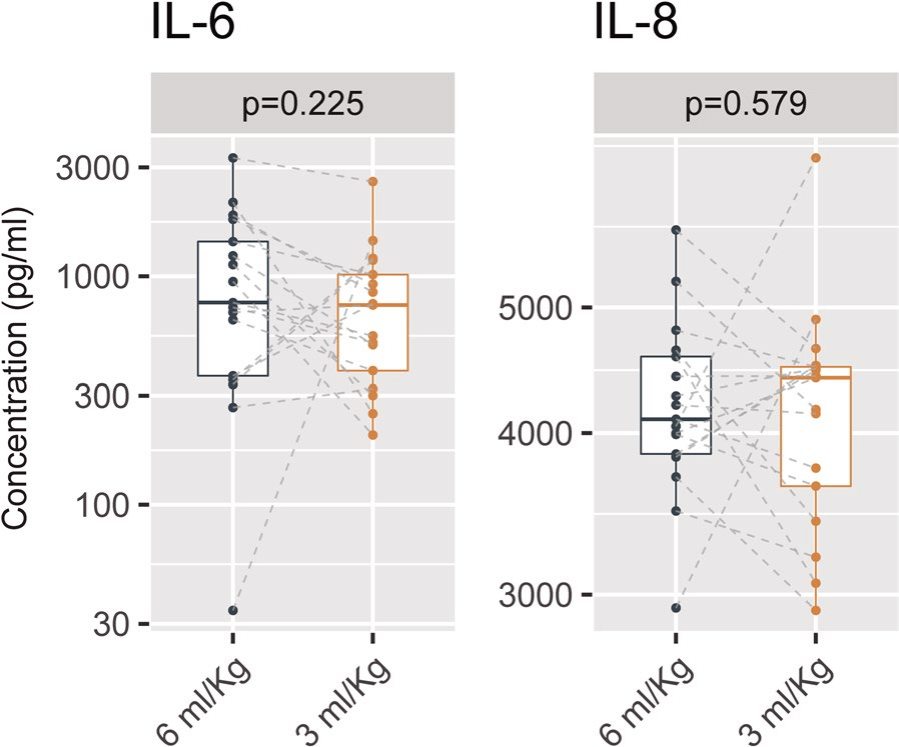
Concentration of IL-6 and IL-8 in bronchoalveolar lavage fluid during ventilation with a tidal volume of 6 ml/kg PBW or 3 ml/kg PBW. Dots and dashed lines show the individual values for each patient. *Y*-axis is traced using a logarithmic scale. The lower and upper hinges correspond to the first and third quartiles (the 25th and 75th percentiles). The upper and lower whiskers extend from the hinge to the largest or smallest value no further than 1.5 times the interquartile range from the hinge. Individual values are shown as points. Values for a given patient are connected by dashed lines. *P* values were obtained using a Wilcoxon test for paired data. *PBW* predicted body weight, *IL* interleukin

Overall, concentration of cytokines in BALF was not modified by the ventilatory settings (Additional file 2: Figure S2). Figure 2 shows the box plots and individual values of IL6 and IL8 for each ventilatory strategy. Cluster analysis of the differences in cytokine levels between ventilation with 6 and 3 ml/kg PBW revealed four groups of mediators with similar behaviour (Additional file 2: Figure S3).

Several exploratory analyses were performed to better characterize the inflammatory response to ventilation. First, changes in inflammatory mediators in patients with high and low driving pressures during 6 ml/kg PBW ventilation (using a threshold of 15 cmH_2_O) were compared. No significant differences were observed in any of the analysed mediators between both groups (Additional file 2: Figure S4). The use of median driving pressure (13 cmH_2_O) as threshold did not yield any significant differences (data not shown). Similarly, there were no differences in these analytes between patients with high and low plateau pressures with the same ventilatory strategy (using the median value, 19 cmH_2_O, as threshold, Additional file 2: Figure S5).

Eleven out of 17 patients were classified as hyperinflamed. The proportion of hyperinflamed patients was similar in those who were ventilated first with 6 ml/kg and those receiving a tidal volume of 3 ml/kg PBW (5 out of 11 and 3 out of 6, respectively, Fisher test *p* = 1). Plotting BALF IL-6 concentration against the study day argued against a reduction of this mediator over time, suggesting that the observed differences are caused by the inflammatory profile and ventilatory strategy, but not the course of the disease itself (Additional file 2: Figure S6).

**Fig. 3:**
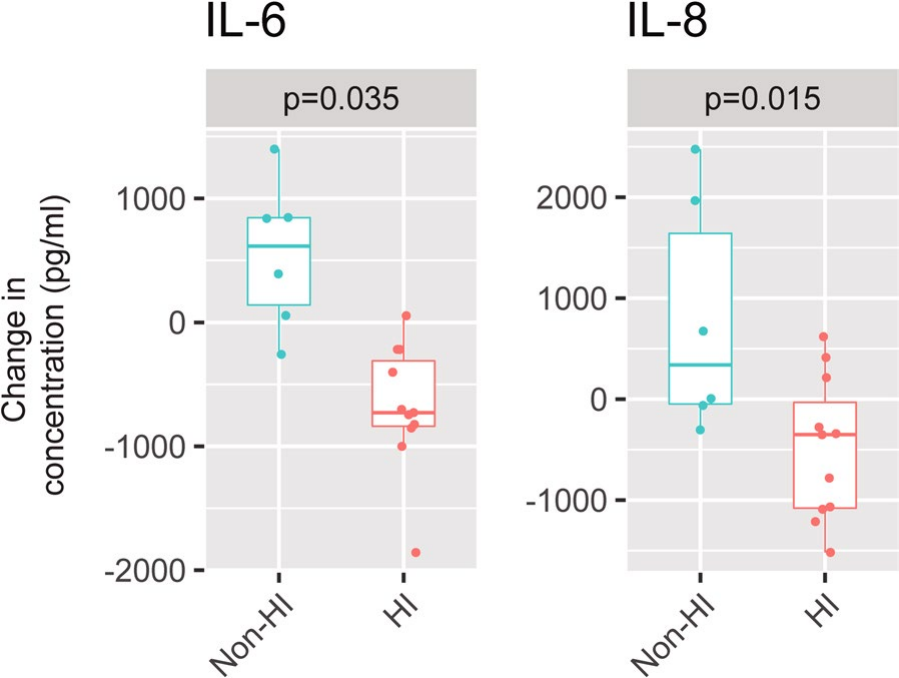
Change in IL-6 and IL-8 from ventilation with 6 ml/kg predicted body weight (PBW) to 3 ml/kg PBW in hyperinflamed and non-hyperinflamed patients (defined using a threshold in IL-6 levels during ventilation with 6 ml/kg PBW of 680 pg/ml). The lower and upper hinges correspond to the first and third quartiles (the 25th and 75th percentiles). The upper and lower whiskers extend from the hinge to the largest or smallest value no further than 1.5 times the interquartile range from the hinge. Individual values are shown as points. *P* values were obtained using an analysis of covariance (ANCOVA) test. *IL* interleukin

There were no differences in age, sex and severity between hyperinflamed and non-hyperinflamed patients (Table 2). There were no differences in ventilatory parameters, respiratory mechanics or haemodynamic parameters between hyperinflamed and non-hyperinflamed patients (Table 2, Additional file 2: Table S3). Fluid balance was compared between inflammatory profiles and ventilatory strategies, with no significant differences being found (Table 2). Changes in driving pressure after decreasing tidal volumes from 6 to 3 ml/kg PBW were 3 (3–4.5) and 6 (4–7) cmH_2_O in hyperinflamed and non-hyperinflamed patients, respectively (*p* = 0.65 in the ANCOVA). The difference in cytokine levels between ventilation with 6 and 3 ml/kg PBW was compared between hyperinflamed and non-hyperinflamed patients. As shown in Fig. 3, there were significant differences between these groups regarding the change in IL-6 and IL-8, as these mediators decreased after reducing tidal volume only in hyperinflamed patients, and increased in the non-hyperinflamed group. There was a trend to similar differences in IL-17A and MCP-1 (Additional file 2: Figure S7).

**Table 2:** Comparison between patients with and without a hyperinflammatory subtype (defined as a IL-6 level in bronchoalveolar lavage fluid above 680 pg/ml during ventilation with a tidal volume of 6 ml/kg)

	Hyperinflamed	Non-hyperinflamed	*P*
Age	58 (54–63)	62 (47–66)	0.840
Sex	7 male/4 female	5 male/1 female	0.768
SAPS3	58 (53–74)	57 (62–69)	0.725
Days on ECMO	9 (6–15)	8 (7–9)	0.880
Days of mechanical ventilation	14 (8–29)	18 (10–20)	0.960
Mortality	6 (55%)	5 (83%)	0.333
DP (cmH_2_O) at 6 ml/kg PBW	10 (10–11)	13 (12–14)	0.189
DP (cmH_2_O) at 3 ml/kg PBW	7 (7–9)	7 (6–9)	1
RR (min^−1^) at 6 ml/kg PBW	12 (10–13)	12 (11–14)	0.473
RR (min^−1^) at 3 ml/kg PBW	11 (10–14)	13 (11–14)	0.444
PaO_2_ (mmHg) at 6 ml/kg PBW	104 (90–117)	92 (75–101)	0.264
PaO_2_ (mmHg) at 3 ml/kg PBW	105 (83–170)	83 (72–110)	0.350
pH at 6 ml/kg PBW	7.45 (7.43–7.50)	7.45 (7.43–7.48)	0.827
pH at 3 ml/kg PBW	7.40 (7.34–7.45)	7.40 (7.37–7.45)	0.879
HR (min^−1^) at 6 ml/kg PBW	87 (79–98)	86 (84–88)	0.664
HR (min^−1^) at 3 ml/kg PBW	88 (76–91)	93 (92–95)	0.664
PCP (mmHg) at 6 ml/kg PBW	14 (12–19)	17 (16–18)	0.694
PCP (mmHg) at 3 ml/kg PBW	18 (12–23)	14 (13–14)	0.793
MAP (mmHg) at 6 ml/kg PBW	76 (71–84)	74 (66–83)	0.828
MAP (mmHg) at 3 ml/kg PBW	74 (68–90)	70 (68–75)	0.545
Fluid balance at 6 ml/kg (l)	1.50 (-0.59–3.60)	0.46 (0.04–1.60)	0.660
Fluid balance at 3 ml/kg (l)	2.08 (0.79–4.34)	1.14 (0.61–1.63)	0.462

Data are expressed as median (interquartile range)

*SAPS3:* Simplified Acute Physiology Score III, *DP:* driving pressure, *PBW:* predicted body weight, *RR:* respiratory rate, *HR:* heart rate, *MAP:* mean systemic arterial pressure

**Fig. 4:**
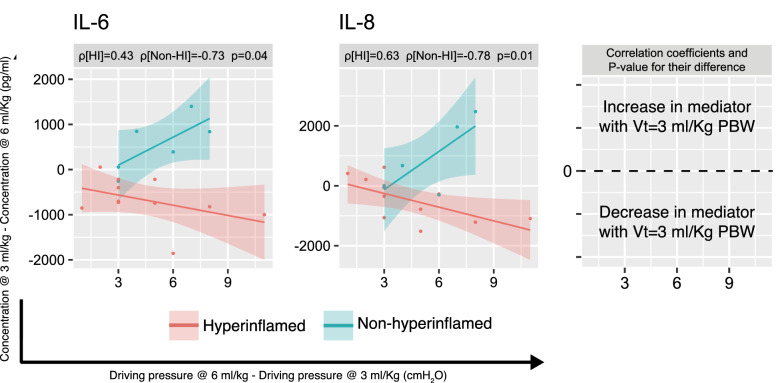
Correlation between the change in IL-6 and IL-8 BALF levels from ventilation with 6 ml/kg predicted body weight (PBW) to 3 ml/kg PBW and the corresponding change in driving pressure in hyperinflamed and non-hyperinflamed patients (defined using a threshold in IL-6 levels during ventilation with 6 ml/kg PBW of 680 pg/ml). Note that the *x*-axis represents the change in driving pressure when tidal volume is decreased, which is mainly defined by compliance of the respiratory system. *IL* interleukin. *P* values correspond to the comparison between the two correlation coefficients

Overall, changes in the inflammatory mediators were not significantly correlated with the change in driving pressure (Additional file 2: Figure S8). However, when the baseline inflammatory response was taken into account, hyperinflamed and non-hyperinflamed patients exhibited a significantly different pattern. In hyperinflamed patients, the reduction in driving pressure achieved by lowering tidal volume was accompanied by a linear decrease in IL-6, IL-8 (Fig. 4) and a group of mediators (IFN-γ, IL-1β, IL-12p70; Additional file 2: Figure S9). Conversely, in non-hyperinflamed patients, the reduction in driving pressure with Vt of 3 ml/kg PBW was correlated with an increase in these group of mediators.

## Discussion

Our results show that ultra-low tidal volume ventilation with 3 ml/kg PBW for 24 h in patients requiring V–A ECMO due to cardiogenic shock was associated with a reduction in lung mechanical load but not with significant changes in the local inflammatory response. In post hoc analyses, we found that patients with a proinflammatory response during conventional ventilation (Vt = 6 ml/kg, PBW) showed lower cytokines levels, proportional to the decrease in driving pressure achieved with ultra-low tidal volume ventilation. Conversely, those without a proinflammatory profile, showed the opposite behaviour (Fig. 4, Additional file 2: Figure S8).

Low tidal volume ventilation is the mainstay of supportive treatment in patients with ARDS [19], and could minimize lung damage in organ donors [20], before lung transplantation [21] or during major surgery [22]. Ultraprotective ventilation in this setting, supported by CO_2_ removal or ECMO, may help to reduce alveolar inflammation [23]. However, the extension of this recommendation to other diseases is not straightforward. A recent trial demonstrated the safety of moderate volumes in patients with normal lungs [24]. In the special case of CPE, tidal volumes of 6–10 ml/kg PBW have been recommended [25], based on a retrospective report showing an increase in mortality with tidal volumes above 9.3 ml/kg PBW [26].

Traditional understanding of the Starling’s mechanisms involved in the development of CPE rationalized this syndrome as non-inflammatory [27]. However, there are several mechanisms that could predispose lungs with CPE to injury. First, there is increasing evidence of proinflammatory responses in these patients. Increased hydrostatic pressures may enhance inflammation [28], and patients with cardiogenic pulmonary oedema show a proinflammatory monocyte profile [29]. Moreover, there is evidence that cardiogenic shock may be associated with alveolocapillary barrier damage and inflammation [30], with an increase in BALF cytokine concentrations above the low levels reported in cardiogenic pulmonary oedema [31]. Second, the observed reduction in EELV caused by alveolar flooding may contribute to a heterogeneous distribution of the ventilation with a baby-lung effect [32], similar to that observed in patients with ARDS. In this setting, mechanical ventilation with conventional tidal volumes increases lung strain and regional overdistension in aerated areas, facilitating the development of VALI. In addition, changes in tidal volume may affect ventricular pre and after loads thus modifying native cardiac output [33]. Conversely, reduction of tidal volumes leads to further lung collapse, as shown by the low EELV during ventilation with a tidal volume of 3 ml/kg PBW, potentially leading to further impairments in respiratory mechanics, gas exchange and haemodynamic [34]. ECMO may help to maintain normal PaCO_2_ by adjusting sweep gas flow. Of note, PEEP levels (which were left at clinicians’ discretion per protocol) were higher during ventilation with ultralow tidal volumes. Although the impact of this difference in PEEP on the observed results cannot be isolated, keeping PEEP constant could have led to even lower EELVs and an even higher risk of injury due to low volume ventilation.

ARDS subphenotypes with different inflammatory response and clinical outcomes [3] point to the necessity of individualized approaches. There were no significant differences in clinical data between hyperinflamed and non-hyperinflamed patients, highlighting the need for specific strategies or biomarkers in critically ill patients that could help to characterize these subtypes [35]. Other authors have suggested that, in addition to compliance, dead space measurements could help to identify those patients in which extracorporeal CO_2_ removal could lead to significant decreases in driving pressures [36]. However, bedside measurements or estimations of dead space in the presence of an established CO_2_ removal device may be difficult to obtain, and their impact on hyperinflamed or non-hyperinflamed patients has not been determined.

We measured a set of immune mediators to give a broad view of the inflammatory response in these patients. However, we cannot discard other specific biomarkers would yield different results, as the lung biological response to ventilation includes a wide range of cellular pathways with specific markers from epithelial/endothelial injury to inflammation, apoptosis or matrix remodelling [37]. Rather than identifying specific molecular mechanisms, our results illustrate the differential response to ventilation. CPE may require different ventilatory adjustments depending not only on respiratory mechanics but also on baseline biological state. In this study, hyperinflamed patients showed significantly lower cytokine levels when placed on Vt 3 ml/kg PBW, compared to the cytokine levels obtained on Vt 6 ml/kg PBW, suggesting attenuated VALI when the ultra-low tidal volume strategy was applied. Conversely, non-hyperinflamed patients exhibited higher proinflammatory mediators’ levels during ventilation with Vt 3 ml/kg PBW. These changes were correlated with the magnitude of decrease of transpulmonary pressure, and could be explained by the predominance of overdistension or atelectrauma. However, it is unclear if these mediators play a pathogenetic role or are biomarkers of ongoing repair [38]. Of note, in both hyperinflamed and non-hyperinflamed patients none of the haemodynamic parameters were significantly modified by the change in tidal volumes. Collectively, these results suggest the existence of underlying, inflammation-driven subtypes that determine the response to ventilation.

The translation of these hypothesis-generating findings may have a relevant clinical impact, to either apply or avoid ultra-low tidal volume ventilation depending on the inflammatory profile. However, further studies addressing this hypothesis are required to optimize and stratify clinical management.

Our study has several limitations. First, the crossover design is prone to carry-over effects. The trial was done without a washout period to minimize the impact of the evolution of the disease on our measurements. However, it has been shown that two or more hours of ventilation cause a change in the inflammatory profile of the lung [39] and we did not find a difference between patients with different initial ventilatory strategy. This crossover design was chosen to achieve the target sample size in a reasonable period, as recruitment of large samples of ECMO patients may be a challenge [40], and the purpose of this study was exploratory. Second, no baseline measurements were performed to avoid an additional invasive procedure such as the bronchoalveolar lavage. However, half of the patients were ventilated first with 6 ml/kg PBW (which could be considered baseline conditions) and we observed no effect caused by the order of application of tidal volumes. Third, the observed effect size of tidal volume in BALF IL-6 levels and its variability were higher than the expected values used for sample size calculation. Therefore, the study may be underpowered to detect a relevant change in IL-6 (or other mediators) in an unselected population. Fourth, the heterogenous behaviour of patients with CPE requires large sample sizes to achieve enough statistical power to demonstrate a difference in patient-centred outcomes such as mortality. We did not observe a significant change in SOFA scores with ventilation. However, SOFA scores have a poor performance to predict mortality in V–A ECMO patients, unless data on right ventricle function are added to the score [41]. Rather, our results may help to identify a subgroup of patients which could benefit from a reduction of tidal volume, thus facilitating the inclusion of an enriched population in clinical trials.

## Conclusion

Collectively, our findings suggest the existence of VALI in patients with CPE even though airway pressures were below the currently accepted safety thresholds. In these settings, ultra-low tidal volume ventilation is feasible. Post hoc analyses suggest that a subgroup of patients with hyperinflamed lungs may benefit from an ultraprotective approach to ventilation, facilitated by extracorporeal support. Our findings highlight the need of appropriate biomarkers that may allow clinicians to anticipate the response to a specific therapeutic approach [42].

## Supplementary Information

**Additional file 1. ** Online supplementary material.

**Additional file 2: Table S1.** Online supplementary results.

## Data Availability

All datasets underlying the manuscript results are available upon request to the corresponding author (LAR). All of the individual participant data collected during the trial, informed consent forms, study protocol, statistical analysis plan, analytic R code and raw data, are available under reasonable request, and after de-identification.

## Abbreviations

ANCOVA: Analysis of covariance
ARDS: Acute respiratory distress syndrome
BALF: Bronchoalveolar lavage fluid
CPE: Cardiogenic pulmonary oedema
ECMO: Extracorporeal membrane oxygenation
EELV: End-expiratory lung volume
FRC: Functional residual capacity
ICU: Intensive care unit
IFN: Interferon
IL: Interleukin
MCP: Monocyte chemoattractant protein
PEEP: Positive end-expiratory pressure
TNF: Tumour necrosis factor
V–A ECMO: Venous–arterial extracorporeal membrane oxygenation
VALI: Ventilator-associated lung injury
Vt: Tidal volume
vv-ECMO: Veno-venous extracorporeal membrane oxygenation
