# Supplementary material for: Biotrauma during ultra-low tidal volume ventilation and venoarterial extracorporeal membrane oxygenation in cardiogenic shock: a randomized crossover clinical trial
Source: Ann Intensive Care. 2021 Aug 28;11:132. doi: 10.1186/s13613-021-00919-0 (PMC8397875; doi:10.1186/s13613-021-00919-0)
Supplement: Supplementary file 2 — Additional file 2: Table S1. Online supplementary results. [file 13613_2021_919_MOESM2_ESM.docx]

**Biotrauma during ultra-low tidal volume ventilation and venoarterial extracorporeal membrane oxygenation in cardiogenic shock: a randomized cross-over clinical trial.**

Laura Amado-Rodríguez MD, PhD, Cecilia Del Busto MD, Inés López-Alonso PhD, Diego Parra MD, PhD, Juan Mayordomo-Colunga MD, PhD, Miguel Arias-Guillén MD, PhD, Rodrigo Albillos-Almaraz MD, Paula Martín-Vicente MSc, Cecilia López-Martínez MSc, Covadonga Huidobro PhD, Luigi Camporota MD, Arthur S Slutsky MD, Guillermo M Albaiceta MD, PhD

**Additional file 2**

**Table of contents**

**Table S1**. Clinical differences between patients randomized to receive first a tidal volume of 6 or 3 ml/Kg predicted body weight (PBW).

**Table S2.** Hemodynamic parameters during ventilation with tidal volumes of 6 and 3 ml/kg predicted body weight (PBW).

**Table S3.** Differences between patients with and without a hyperinflammatory subtype (defined as a IL-6 level in bronchoalveolar lavage fluid above 680 pg/ml during ventilation with a tidal volume of 6 ml/Kg predicted body weight). Data are expressed as median (interquartile range).

**Figure S1.** Identification of a IL-6 threshold.

**Figure S2.** Concentration of inflammatory mediators in bronchoalveolar lavage fluid during ventilation with a tidal volume of 6 ml/Kg predicted body weight (PBW) or 3 ml/Kg PBW.

**Figure S3**. Clustering of inflammatory mediators.

**Figure S4.** Changes in inflammatory mediators according to baseline driving pressure (DP).

**Figure S5.** Changes in inflammatory mediators according to baseline plateau pressure (Pplat).

**Figure S6.** Interleukin-6 concentration in each study day.

**Figure S7.** Change in inflammatory mediators from ventilation with 6 ml/Kg predicted body weight (PBW) to 3 ml/Kg PBW in hyperinflamed and non-hyperinflamed patients (defined using a threshold in IL-6 levels during ventilation with 6 ml/Kg PBW of 680 pg/ml).

**Figure S8**. Correlation between changes in driving pressures and changes in inflammatory mediators.

**Figure S9**. Correlation between the change in inflammatory mediators from ventilation with 6 ml/Kg predicted body weight (PBW) to 3 ml/Kg PBW and the corresponding change in driving pressure in hyperinflamed and non-hyperinflamed patients (defined using a threshold in IL-6 levels during ventilation with 6 ml/Kg PBW of 680 pg/ml).

**Table S1**. Clinical differences between patients randomized to receive first a tidal volume of 6 or 3 ml/Kg predicted body weight (PBW). BMI: body mass index. HT: hypertension. DM: diabetes mellitus.

|  | 6 ml/Kg PBW | 3 ml/Kg PBW | p |
| --- | --- | --- | --- |
| Age (years) | 59 (54-64) | 61 (48-68) | 0.883 |
| Sex | 6 male / 4 female | 6 male / 1 female | 0.338 |
| SAPS-3 | 59 (52-73) | 58 (51-68) | 0.769 |
| Cause of shock:  Myocardial infarction  Cardiac arrest after myocardial infarction  Post-surgical  Myocarditis | 4  1  4  1 | 2  2  1  2 | 0.815 |
| BMI | 26.30 (24.68-26.92) | 26.76 (22.25-30.38) | 0.874 |
| Co-morbidities  HT  DM  Tobacco | 6/10  1/10  7/10 | 5/7  0/7  5/7 | 1  1  1 |

**Table S2.** Hemodynamic parameters during ventilation with tidal volumes of 6 and 3 ml/Kg predicted body weight (PBW). Data are expressed as median (interquartile range). Data on right ventricle output and pressures in the pulmonary circuit were available for 11 patients.

|  | 6 ml/Kg PBW | 3 ml/Kg PBW | p |
| --- | --- | --- | --- |
| Heart rate (min^-1^) | 87 (81-94) | 90 (78-93) | 0.027 |
| Right ventricle output (l/min) | 5.1 (2.7-8.3) | 4.9 (2.5-6.8) | 0.496 |
| Mean systemic arterial pressure (mmHg) | 76 (69-84) | 70 (67-77) | 0.500 |
| Mean pulmonary arterial pressure (mmHg) | 24 (18-29) | 26 (19-32) | 0.105 |
| Central venous pressure (mmHg) | 12 (9-14) | 12 (11-17) | 0.774 |
| Pulmonary artery occlusion pressure (mmHg) | 16 (12-18) | 14 (12-22) | 0.943 |
| Arterial lactate (mM) | 1.4 (1.2-1.8) | 1.4 (0.8-2) | 0.917 |

**Table S3.** Differences between patients with and without a hyperinflammatory subtype (defined as a IL-6 level in bronchoalveolar lavage fluid above 680 pg/ml during ventilation with a tidal volume of 6 ml/Kg predicted body weight). Data are expressed as median (interquartile range). SAPS3: Simplified Acute Physiology Score III. ECMO: extracorporeal membrane oxygenation. PBW: predicted body weight.

|  | Hyperinflamed | Non-hyperinflamed | p |
| --- | --- | --- | --- |
| Age | 58 (54-63) | 62 (47-66) | 0.840 |
| Sex | 7 male / 4 female | 5 male /1 female | 0.768 |
| SAPS3 | 58 (53-74) | 57 (62-69) | 0.725 |
| Days on ECMO | 9 (6-15) | 8 (7-9) | 0.880 |
| Days of mechanical ventilation | 14 (8-29) | 18 (10-20) | 0.960 |
| Mortality | 6 (55%) | 5 (83%) | 0.333 |
| Ventilation with 6 ml/Kg PBW |  |  |  |
| Tidal volume (ml) | 410 (400-480) | 400 (372-440) | 0.547 |
| Respiratory rate (min^-1^) | 12 (10-13) | 12 (11-14) | 0.473 |
| Plateau pressure (cmH_2_O) | 18 (17-18) | 19 (18-20) | 0.350 |
| PEEP (cmH_2_O) | 7 (5-8) | 6 (5-6) | 0.403 |
| Driving pressure (cmH_2_O) | 10 (10-11) | 13 (12-14) | 0.189 |
| Respiratory system compliance (ml/cmH_2_O) | 36 (32-43) | 31 (28-36) | 0.429 |
| EELV (ml) | 1043 (664-1088) | 878 (751-931) | 0.413 |
| Strain | 0.54 (0.48-0.61) | 0.43 (0-34-0.57) | 0.286 |
| PaO_2_ (mmHg) | 104 (90-117) | 92 (75-101) | 0.264 |
| PaCO_2_ (mmHg) | 36 (27-37) | 36 (34-38) | 0.713 |
| pH | 7.45 (7.43-7.50) | 7.45 (7.43-7.48) | 0.827 |
| FiO_2_ | 0.40 (0.36-0.55) | 0.40 (0.36-0.48) | 0.955 |
| F_ECMO_O_2_ | 0.70 (0.60-0.80) | 0.60 (0.60-0.71) | 0.692 |
| ECMO blood flow(l/min) | 3.6 (3.0-3.9) | 2.9 (2.8-3.1) | 0.277 |
| ECMO sweep flow (l/min) | 3.3 (3.0-4.8) | 2.3 (2.0-5.9) | 0.380 |
| Heart rate (min^-1^) | 87 (79-98) | 86 (84-88) | 0.664 |
| Right ventricle output (l/min) | 4.7 (2.4-7.4) | 6.3 (4.5-8.5) | 0.630 |
| Mean systemic arterial pressure (mmHg) | 76 (71-84) | 74 (66-83) | 0.828 |
| Mean pulmonary arterial pressure (mmHg) | 25 (17-32) | 19 (19-23) | 0.919 |
| Central venous pressure (mmHg) | 12 (10-16) | 10 (9-11) | 0.287 |
| Pulmonary capillary pressure (mmHg) | 14 (12-19) | 17 (16-18) | 0.694 |
| Arterial lactate (mM) | 1.6 (1.2-2.3) | 1.3 (1-1.4) | 0.276 |
| Ventilation with 3 ml/Kg PBW |  |  |  |
| Tidal volume (ml) | 220 (185-223) | 207 (200-234) | 0.840 |
| Respiratory rate (min^-1^) | 11 (10-14) | 13 (11-14) | 0.444 |
| Plateau pressure (cmH_2_O) | 15 (12-16) | 16 (14-17) | 0.418 |
| PEEP (cmH_2_O) | 7 (6-8) | 9 (8-10) | 0.055 |
| Driving pressure (cmH_2_O) | 7 (7-9) | 7 (6-9) | 1 |
| Respiratory system compliance (ml/cmH_2_O) | 28 (23-33) | 25 (23-31) | 0.943 |
| EELV (ml) | 538 (494-820) | 666 (416-833) | 1 |
| Strain | 0.38 (0.22-0.42) | 0.39 (0.28-0.62) | 0.556 |
| PaO_2_ (mmHg) | 105 (83-170) | 83 (72-110) | 0.350 |
| PaCO_2_ (mmHg) | 39 (31-42) | 40 (39-44) | 0.350 |
| pH | 7.40 (7.34-7.45) | 7.40 (7.37-7.45) | 0.879 |
| FiO_2_ | 0.40 (0.38-0.50) | 0.40 (0.36-0.44) | 0.836 |
| F_ECMO_O_2_ | 0.70 (0.55-0.75) | 0.70 (0.63-0.78) | 0.410 |
| ECMO blood flow(l/min) | 3.3 (3.2-3.5) | 3.1 (2.7-3.5) | 0.880 |
| ECMO sweep flow (l/min) | 5 (3-6) | 5 (4-7) | 0.579 |
| Heart rate (min^-1^) | 88 (76-91) | 93 (92-95) | 0.664 |
| Right ventricle output (l/min) | 3.6 (2.2-7.6) | 5.9 (5.7-6) | 0.694 |
| Mean systemic arterial pressure (mmHg) | 74 (68-90) | 70 (68-75) | 0.545 |
| Mean pulmonary arterial pressure (mmHg) | 26 (18-31) | 29 (26-31) | 0.723 |
| Central venous pressure (mmHg) | 13 (11-18) | 11 (10-12) | 0.162 |
| Pulmonary capillary pressure (mmHg) | 18 (12-23) | 14 (13-14) | 0.793 |
| Arterial lactate (mM) | 1.4 (0.9-2.5) | 1.4 (0.9-1.8) | 0.801 |

**Figure S1.** Identification of a IL-6 threshold**.**

Concentrations of IL-6 in bronchoalveolar lavage from ARDS patients included in a previously published article (1) were analyzed to identify the optimal threshold value to distinguish those with a proinflammatory lung response to ventilation. From data inspection, there is substantial evidence of an IL-6 threshold that differentiates patients with ongoing inflammation, compared to both ARDS patients without inflammation or patients with no ARDS. Panel A shows the ROC curve of IL-6 levels to identify patients with a pro-inflammatory cytokine profile in bronchoalveolar lavage fluid in response to high strain levels. Panel B plots accuracy ([True positives + True negatives] / [Positives+Negatives]) against IL-6 levels. From this analysis, a BALF IL-6 concentration of 680 pg/ml was the optimal value to identify that population.

**Figure S2.** Concentration of inflammatory mediators in bronchoalveolar lavage fluid during ventilation with a tidal volume of 6 ml/Kg predicted body weight (PBW) or 3 ml/Kg PBW.

Dots and dashed lines show the individual values for each patient. Y-axis is traced using a logarithmic scale. The lower and upper hinges correspond to the first and third quartiles (the 25^th^ and 75^th^ percentiles). The upper and lower whiskers extend from the hinge to the largest or smallest value no further than 1.5 times que interquartile range from the hinge. Individual values are shown as points. Values for a given patient are connected by dashed lines. P values were obtained using a Wilcoxon test for paired data.


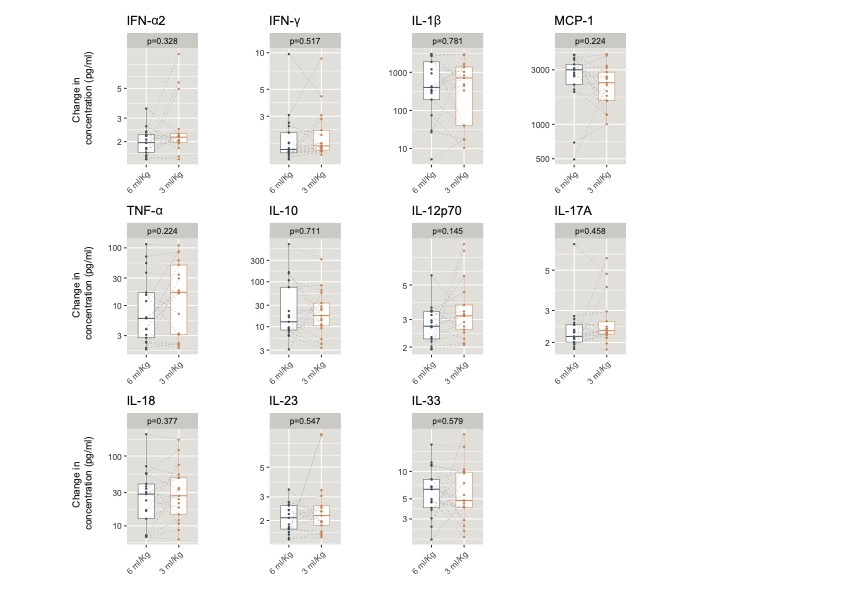


**Figure S3.** Representation of the hierarchical clustering and heatmap showing the correlation coefficients for the change between ventilation with 6 and 3 ml/Kg predicted body weight, for each pair of measured cytokines. Asterisks mark correlations with a p value below 0.05.


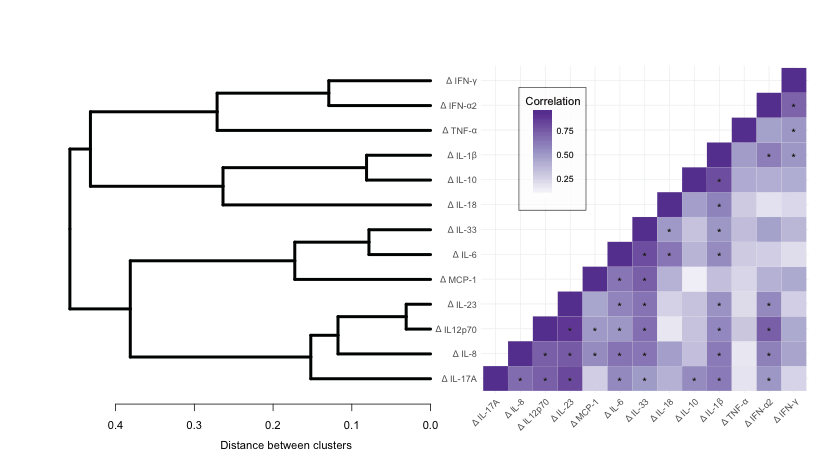


**Figure S4**. Changes in inflammatory mediators according to baseline driving pressure (DP).

The differences in bronchoalveolar mediators between ventilation with tidal volume of 6 and 3 ml/Kg predicted body weight were compared according to the baseline DP (this is, during ventilation with a tidal volume of 6 ml/Kg), using a previously published threshold of 15 cmH_2_O (2). There were no differences in any mediator between the two groups. P-values were calculated using an analysis of covariance (ANCOVA).

**Figure S5.** Changes in inflammatory mediators according to baseline plateau pressure (Pplat).

The differences in bronchoalveolar mediators between ventilation with tidal volume of 6 and 3 ml/Kg predicted body weight (PBW) were compared according to the baseline Pplat (this is, during ventilation with a tidal volume of 6 ml/Kg PBW), using as threshold the median value of the overall sample (19 cmH_2_O). There were no differences in any mediator between the two groups. P-values were calculated using an analysis of covariance (ANCOVA).

**Figure S6.** Interleukin-6 concentration in each study day. To discard a time-dependent effect, caused by the disease course, IL-6 levels were plotted against the study day independently of the initial ventilatory strategy. The plot demonstrates that there is not a constant time effect over IL-6 concentration, and that changes are driven by the patient subtype (hyperinflammed or non-hyperinflammed) and the ventilatory strategy.


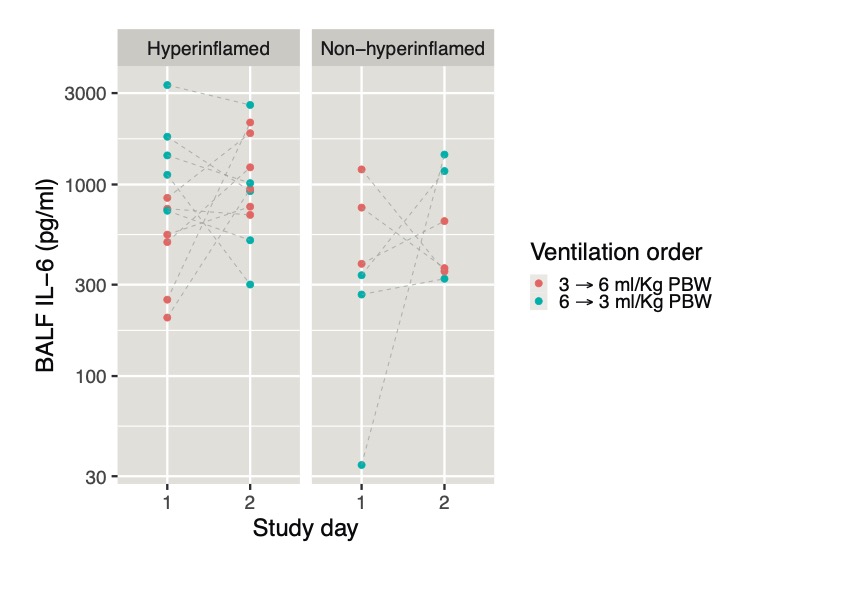


**Figure S7.** Change in inflammatory mediators from ventilation with 6 ml/Kg predicted body weight (PBW) to 3 ml/Kg PBW in hyperinflamed and non-hyperinflamed patients (defined using a threshold in IL-6 levels during ventilation with 6 ml/Kg PBW of 680 pg/ml). The lower and upper hinges correspond to the first and third quartiles (the 25^th^ and 75^th^ percentiles). The upper and lower whiskers extend from the hinge to the largest or smallest value no further than 1.5 times the interquartile range from the hinge. Individual values are shown as points. P-values were obtained using an analysis of covariance (ANCOVA) test.

**Figure S8**. Correlation between changes in driving pressures and changes in inflammatory mediators.

The difference in driving pressure during ventilation with tidal volumes of 6 and 3 ml/kg predicted body weight and the change in inflammatory mediators in the bronchoalveolar lavage fluid were analyzed using linear regression in the whole sample. None of the mediators was linearly correlated with the change in driving pressure.


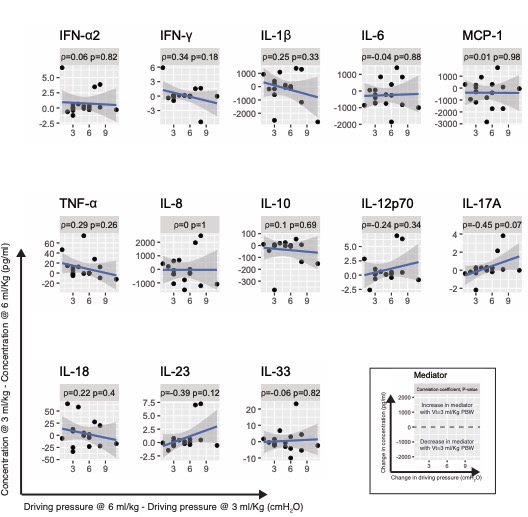


**Figure S9**. Correlation between the change in inflammatory mediators from ventilation with 6 ml/Kg predicted body weight (PBW) to 3 ml/Kg PBW and the corresponding change in driving pressure in hyperinflamed and non-hyperinflamed patients (defined using a threshold in IL-6 levels during ventilation with 6 ml/Kg PBW of 680 pg/ml). Note that the x-axis, represents the change in driving pressure when tidal volume is decreased, which is mainly defined by compliance of the respiratory system. P-values correspond to the comparison between the two correlation coefficients.


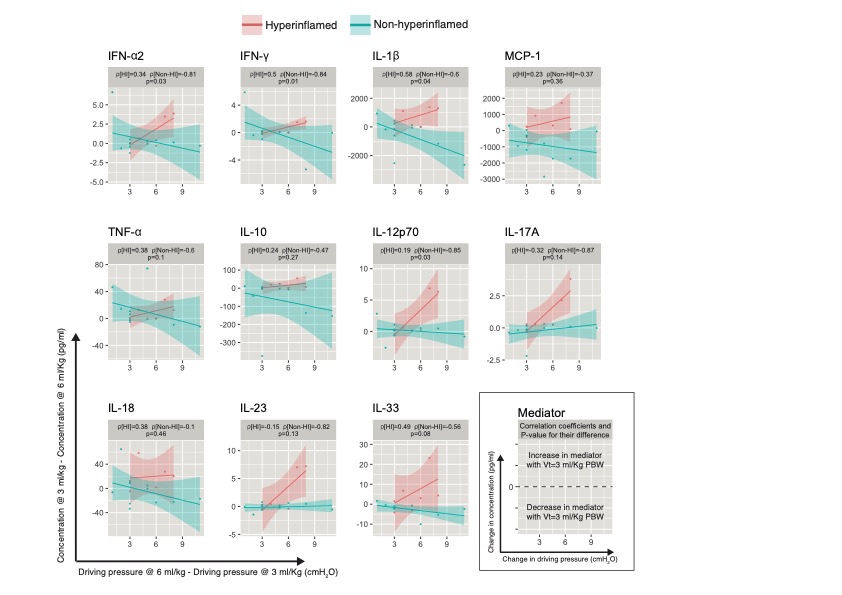


**References**

1. Gonzalez-Lopez A, Garcia-Prieto E, Batalla-Solis E, et al.: Lung strain and biological response in mechanically ventilated patients. *Intensive Care Med* 2012; 38:240–7

2. Amato MB, Meade MO, Slutsky AS, et al.: Driving pressure and survival in the acute respiratory distress syndrome. *N Engl J Med* 2015; 372:747–55
